# Tuning fluorocarbon adsorption in new isoreticular porous coordination frameworks for heat transformation applications†
†Electronic supplementary information (ESI) available: Experimental section, PXRD patterns, crystallographic tables and characterization details, and X-ray crystallographic files in CIF format. CCDC 1031873 and 1031874. For ESI and crystallographic data in CIF or other electronic format see DOI: 10.1039/c4sc03985h



**DOI:** 10.1039/c4sc03985h

**Published:** 2015-02-18

**Authors:** Rui-Biao Lin, Tai-Yang Li, Hao-Long Zhou, Chun-Ting He, Jie-Peng Zhang, Xiao-Ming Chen

**Affiliations:** a MOE Key Laboratory of Bioinorganic and Synthetic Chemistry , School of Chemistry and Chemical Engineering , Sun Yat-Sen University , Guangzhou 510275 , P. R. China . Email: zhangjp7@mail.sysu.edu.cn

## Abstract

Adsorption heat transformation is one of the most energy-efficient technologies, which relies much on the type and performance of the adsorbent–adsorbate pair. Here, we report adsorption behaviors of a typical fluorocarbon R22 (CHClF_2_) in a new series of isoreticular porous coordination polymers [Zn_4_O(bpz)_2_(ldc)], in which the typical Zn_4_O clusters are connected by hydrophobic 3,3′,5,5′-tetramethyl-4,4′-bipyrazolate (bpz^2–^) and different linear dicarboxylates (ldc^2–^) to form non-interpenetrated **pcu** networks with variable pore sizes, shapes, and volumes. Fluorocarbon sorption measurements of these materials revealed high R22 uptakes of 0.73–0.97 g g^–1^ (0.62–0.65 g cm^–3^) at 298 K and 1 bar and working capacities of 0.41–0.72 g g^–1^ (0.35–0.47 g cm^–3^) between 273 and 313 K at about 0.13, 0.11 and 0.52 bar, respectively, as well as very large diffusion coefficients of 5.1–7.3 × 10^–7^ cm^2^ s^–1^. Noteworthily, the R22 sorption performance can be dramatically improved by subtle modification of the pore size and shape, demonstrating porous coordination polymer–fluorocarbon as a promising adsorbent–adsorbate pair for heat transformation applications.

## Abstract 2

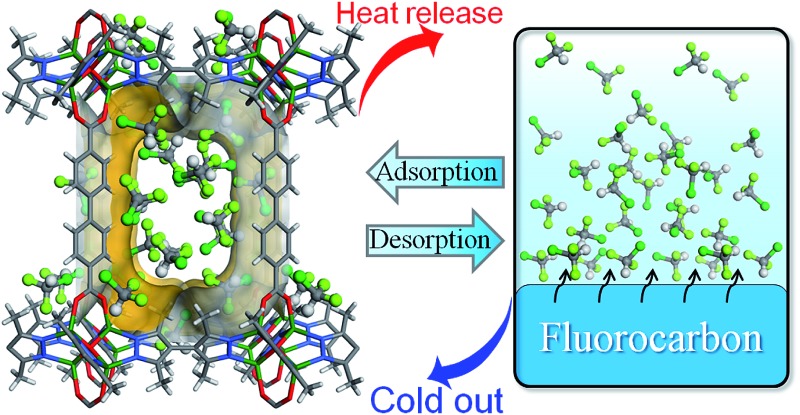
We report adsorption behaviors of a typical fluorocarbon R22 (CHClF_2_) in a new series of isoreticular porous coordination frameworks [Zn_4_O(bpz)_2_(ldc)].

## Introduction

**Scheme 1:**
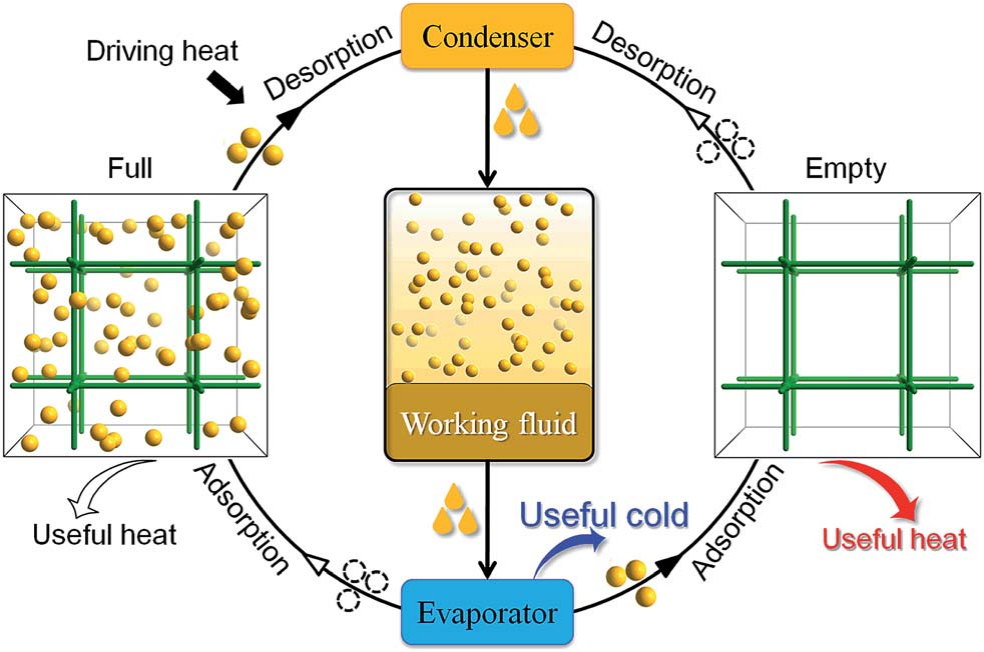
Principle working process of a continuous heat transformation system. In the cold production (adsorption) cycle, the working fluid is evaporated in an evaporator, producing desired cold (useful cold) in the application environment. And adsorption of the vapor from the evaporator into the adsorbent (empty), releasing the adsorption heat to the target environment (useful heat) for heat pump application. Then, the adsorbent (full) is heated by a low-temperature heat source (*e.g.*, solar heat, waste heat or gas burner) to start the regeneration (desorption) cycle. The released vapors condense in the condenser and the condensation heat is released to the environment.

The physical adsorption and desorption of guest molecules onto the surface of a solid is accompanied by heat release and absorption. In an adsorptive heat transformation process, evaporation of a working fluid produces desired cold in the cooling application while adsorption of the fluid vapors into the adsorbent releases heat into the environment. After that, low-temperature heat sources (*e.g.* solar heat, waste heat or gas burner) can be used to regenerate the adsorbent and complete the cycle, which is equivalent to the compression in conventional vapor-compression-driven systems (Scheme 1). Such a working process is suitable for low-temperature heat transformation applications such as thermally driven adsorption chillers or adsorption heat pumps. Many kinds of adsorbent–adsorbate pairs, such as activated carbon–methanol, activated carbon–ammonia, silica gel–water, zeolite–water, and so on, have been developed to fulfill specific application conditions.^1,2^ The working capacity of the adsorbent, *i.e.*, the amount of the working fluid adsorbed and desorbed by a given amount of adsorbent during a complete heating–cooling cycle, is proportional with the amount of heat transferred in each cycle.^3^ Traditionally, typical adsorbents such as zeolites^4,5^ and activated carbons suffer from the lack of structural and functional tunability,^6^ hindering modulation of working capacity which directly correlates to their performances in practical applications.

Porous coordination polymers (PCPs) or metal–organic frameworks (MOFs) are emerging as promising adsorbents^7–10^ for their uniform pore structure,^11–17^ high porosity and tunable pore size.^18–22^ Since many PCPs are hydrophilic and can adsorb large amounts of water, these materials have attracted great interest as adsorbents in water-based adsorption heat transformation systems.^3,23–29^ While water is a preferred adsorbate due to its high evaporation enthalpy and being harmless to the environment, its extremely low saturation pressure requires the system to be vacuum-tight and seriously limits the diffusion rate or cooling power.^1^ Also, it is impossible for adsorption cooling systems based on water to produce temperatures below 0 °C, which also limits their applications.^1^ Ammonia is another interesting adsorbate due to its higher evaporation pressures at low temperatures, but it is limited for indoor use because of its high toxicity and corrosion problems. In contrast, although fluorocarbons have relatively low latent heats of vaporization and have environmental concerns, their suitable boiling points and saturation pressures,^30^ as well as high chemical stability, still enable them as the most popular working fluids in conventional heat transformation systems. Actually, some PCPs may adsorb large amounts of fluorocarbons,^31,32^ but there is still no study about the working capacity of any PCP–fluorocarbon heat transformation system. Herein, we demonstrate that very high fluorocarbon adsorption–desorption working capacity can be achieved by rational modulation of the pore size/shape of PCPs.

## Results and discussion

### Synthesis, structure, stability, and porosity

**Fig. 1:**
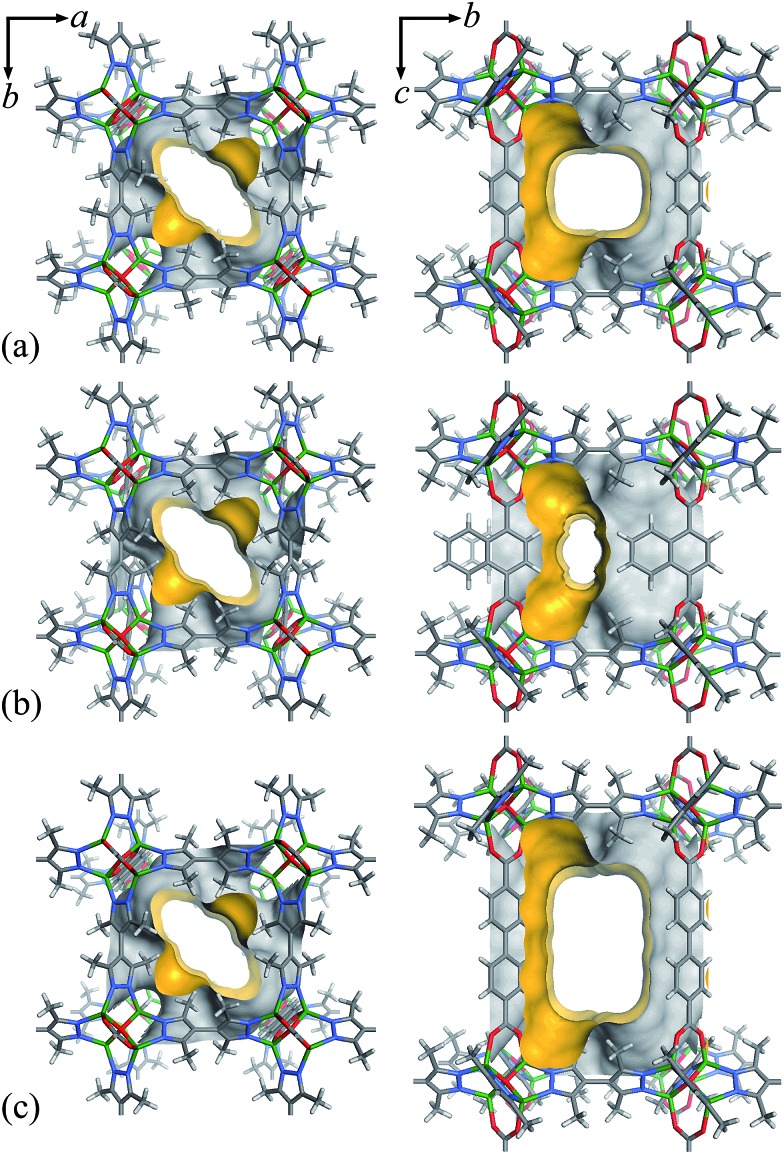
The pore surface structure viewed along two characteristic directions of **1** (a), **2** (b) and **3** (c).

Considering that fluorocarbons are hydrophobic,^33–35^ we selected [Zn_4_O(bpz)_2_(bdc)] (MAF-X10, **1**, H_2_bdc = 1,4-benzenedicarboxylic acid, H_2_bpz = 3,3′,5,5′-tetramethyl-4,4′-bipyrazole, Fig. 1a) as a candidate adsorbent.^36,37^ MAF-X10 is isostructural with [Zn_4_O(bdc)_3_] (MOF-5)^38^ except that two thirds of the bdc^2–^ linkers are substituted by bpz^2–^ linkers, so that the pore surface of MAF-X10 is more hydrophobic and can be use for selective adsorption of some hydrophobic molecules.^39,40^ Moreover, to study the structure–property relationship for fluorocarbon adsorption, we designed and synthesized two new analogs of MAF-X10.

Solvothermal reaction of Zn(NO_3_)_2_, H_2_bpz with naphthalene-1,4-dicarboxylic acid (H_2_ndc) or biphenyl-4,4′-dicarboxylic acid (H_2_bpdc) yielded crystals of two new porous coordination frameworks, namely [Zn_4_O(bpz)_2_(ndc)] (MAF-X12, **2**) and [Zn_4_O(bpz)_2_(bpdc)] (MAF-X13, **3**), respectively (Fig. 1 and S1†). Single-crystal structure analyses confirmed that **2** and **3** are isoreticular with **1**, crystallizing in the same space group *P*4_2_/*mcm* (Table S1†). The coordination frameworks in **1–3** can be all described as non-interpenetrated three-dimensional (3D) **pcu** nets composed of octahedral {Zn_4_O(Rpz)_4_(RCOO)_2_} (Rpz and RCOO denote pyrazolate and carboxylate groups, respectively) cores and two-connected bpz^2–^ and dicarboxylate linkers. In **1–3**, the apertures along the *c*-axis are the same 4.3 × 6.9 Å^2^ since they possess the same bipyrazolate layer across the *ab*-plane. However, since the lengths and side groups of the dicarboxylate ligands are quite different, the pore sizes and shapes of **1–3** vary from each other (Table 1). Since the surface components of **1–3** are exactly the same, their progressively changed pore sizes/shapes should be useful to achieve precisely tunable sorption performance.

**Table 1:** Summary of porosity parameters and comparison of R22 sorption performance for **1–3**

Species	*d*_a_ ^*a*^ [Å^2^]	Cavity [Å^3^]	Void [%]	*V*_c_ ^*b*^ [cm^3^ g^–1^]	*S*_BET_ ^*c*^ [m^2^ g^–1^]	*m*_273_ ^*d*^ [g g^–1^]	*m*_313_ ^*d*^ [g g^–1^]	Δ*m* ^*e*^ [g g^–1^]	*D*_s_ [cm^2^ s^–1^]
**1**	6.6 × 5.8	9.4 × 9.9 × 13.2	63.4	0.798	2032	0.91	0.74	0.46 (0.13 bar)	5.6 × 10^–7^
**2**	3.0 × 5.8	9.4 × 9.9 × 13.2	60.7	0.723	1787	0.82	0.66	0.41 (0.11 bar)	5.1 × 10^–7^
**3**	6.6 × 10.0	9.4 × 9.9 × 15.9	69.5	1.071	2742	1.17	0.73	0.72 (0.52 bar)	7.3 × 10^–7^

^*a*^The apertures sizes along the *a*-axis.

^*b*^The pore volumes estimated from crystal structures.

^*c*^Measured BET surface areas.

^*d*^ *m*_273_ and *m*_313_: R22 uptakes at 273 and 313 K, 1 bar, respectively.

^*e*^Δ*m*: the highest working capacities between 273 and 313 K (at corresponding working pressures).

Thermogravimetry and powder X-ray diffraction (PXRD) showed that **1–3** can completely release all guest molecules at *ca.* 100 °C (Fig. S2 and S3†). The framework decomposition temperatures of **2** and **3** are *ca.* 450 °C, being lower than that of 550 °C for MAF-X10 but still higher than most of other PCPs.^41–43^ The different thermal stabilities of **1–3** can be explained by the different stabilities of the dicarboxylate ligands.^44^

**Fig. 2:**
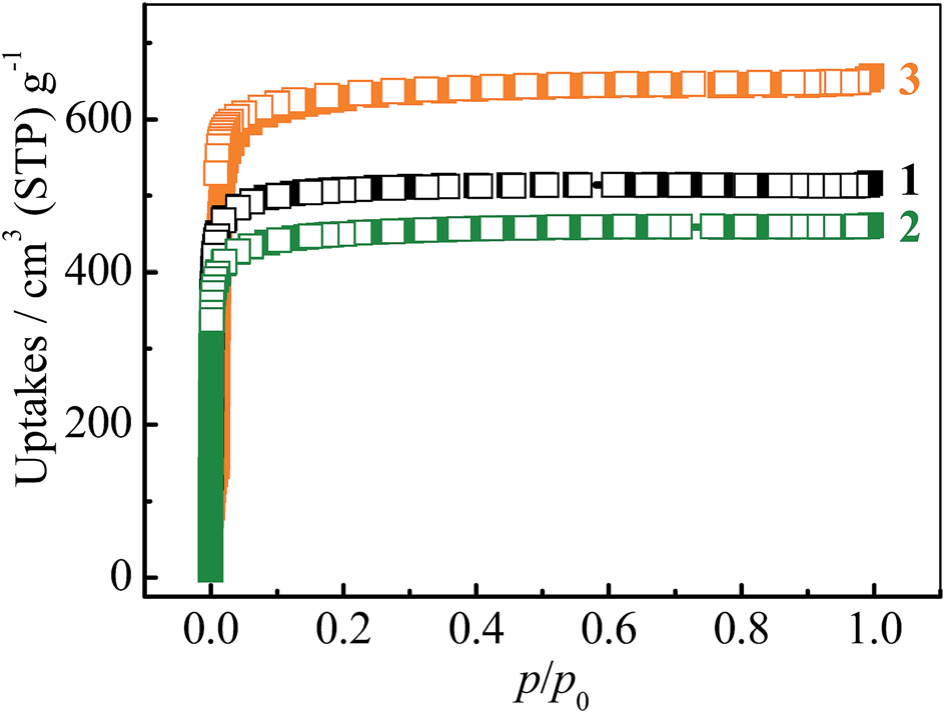
N_2_ adsorption (solid) and desorption (open) isotherms at 77 K for **1–3**.

The N_2_ sorption isotherms of **1–3** measured at 77 K exhibit typical type-I characters with saturated uptakes 516, 461 and 656 cm^3^ (standard temperature and pressure; STP) g^–1^ (Fig. 2 and S4†), corresponding to pore volumes of 0.798, 0.713 and 1.014 cm^3^ g^–1^, respectively, which are close to the values calculated from their crystal structures (Table 1), revealing the high purity and quality of the samples. The Langmuir/BET surface areas of **1–3** are 2239/2032, 2001/1787 and 2838/2742 m^2^ g^–1^, respectively.

### Fluorocarbon adsorption and desorption properties

**Fig. 3:**
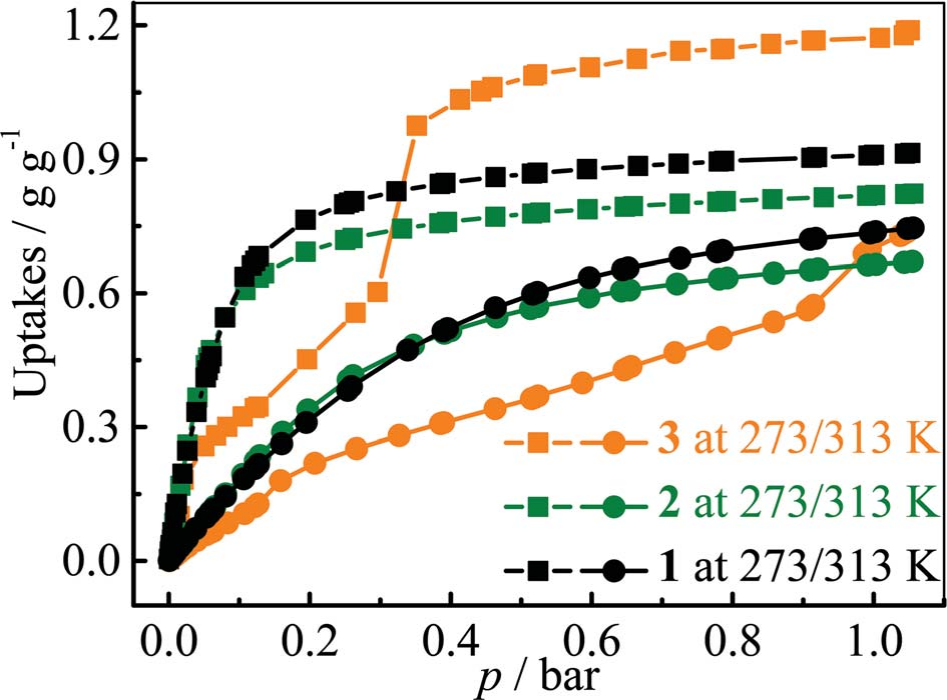
R22 adsorption isotherms measured at 273 (squares) and 313 (circles) K for **1–3**.

The adsorption isotherms for a typical fluorocarbon R22 (CHClF_2_) were measured for **1–3** at ambient conditions (Fig. 3). The sorption isotherms of **1–2** exhibit type-I characters. In the low pressure region, **2** exhibits slightly higher uptake than **1**, but the trend is reversed in the high pressure region. This phenomenon can be explained by using their pore sizes and pore volumes, *i.e.* small and large pores tend to increase the adsorption amounts in the low and high pressure regions, respectively. On the other hand, the R22 isotherm of **3** shows a typical type-IV shape. The isotherms measured at 273 K revealed that the R22 saturation uptake of **3** is much higher than those of **1** and **2**, being consistent with their pore volumes (Table 1). However, due to the unique type-IV isotherm shape, the R22 uptake of **3** in the low pressure region is much lower than those of **1** and **2**. Actually, similar differences can be also found between **3** and other known materials. For example, The R22 uptakes of **3** at room temperature are 0.60 g g^–1^ (0.39 g cm^–3^) and 0.97 g g^–1^ (0.63 g cm^–3^) at 0.6 and 1.0 bar (Fig. S5†), respectively, while those of MIL-101 showing a typical type-I isotherm are 0.66 g g^–1^ (0.41 g cm^–3^) and 0.85 g g^–1^ (0.53 g cm^–3^), respectively.^32^ This special characteristic implies that a relatively large working capacity can be easily achieved by **3** within a narrow range of pressures. At 313 K, the R22 uptakes of **3** are indeed much lower than those of **1** and **2** at a much wider range of pressures, which confirms that the working capacity for **3** in practical applications, *i.e.*, temperature-swing isobaric adsorption–desorption,^45^ should be much higher than those for **1** and **2**. The working capacity at a particular pressure (isobaric adsorption) depends on the difference in uptakes between the lower and higher working temperatures.^23,46–49^ For instance, the highest working capacities for **1–3** between 273 and 313 K can be calculated as 0.46, 0.41, and 0.72 g g^–1^ at about 0.13, 0.11, and 0.52 bar, respectively (Fig. S6†).

**Fig. 4:**
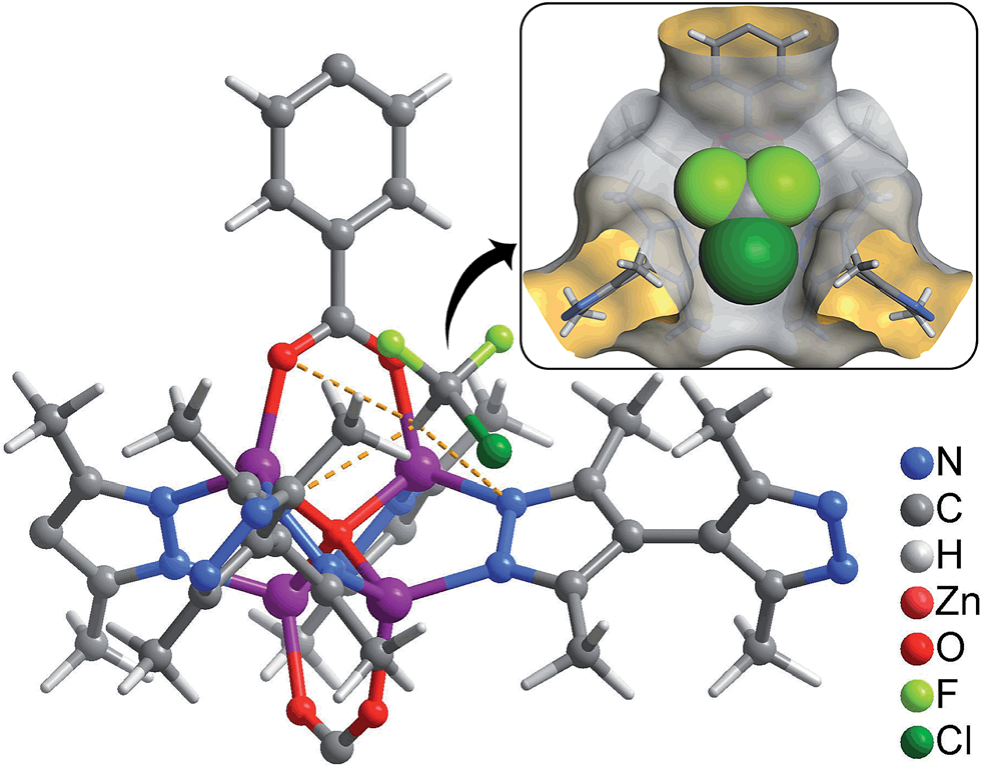
Preferential R22 location in **1** obtained from GCMC calculations (inset: perspective view in space-filling).

Coverage-dependent R22 adsorption enthalpies of **1–3** were calculated using the Clausius–Clapeyron equation using isotherms measured at 273–313 K (Fig. S5 and S7†). The enthalpies of **1–3** at zero coverage are 32.9(1.9), 31.8(1.8), and 31.4(1.1) kJ mol^–1^, respectively. These enthalpies are similar to that of MIL-101 (34.6 kJ mol^–1^)^32^ and higher than those for activated carbon (22.0–28.0 kJ mol^–1^)^46^ and the standard enthalpy of vaporization for R22 (28.2 kJ mol^–1^), which may be associated with the more polar host of PCPs compared with activated carbon. The higher enthalpy means that the system can transfer more heat during the adsorption heat pump processes. The similar zero-coverage adsorption enthalpies indicate that the R22 molecules are initially adsorbed on very similar sites in **1–3**. Actually, the cavity sizes of **1** and **2** are almost identical, while that of **3** is slightly larger, which is consistent with their enthalpy trend. On the other hand, although the aperture sizes are distinct for **1–3**, their surfaces are completely lined by low-polarity C–H moieties, which are not likely the preferential adsorption sites. To explain the similar zero-coverage adsorption enthalpies and identify the primary R22 adsorption sites, the interactions between R22 molecules and **1–3** were investigated by grand canonical Monte Carlo (GCMC) simulations, which showed that the initial binding sites of R22 molecules in **1–3** are almost identical (Fig. 4 and S8†), with binding energies of 33.2, 34.0 and 33.2 kJ mol^–1^, respectively. Interestingly, the R22 molecule lies well on a triangular hydrophobic pocket surrounded by the aromatic face of a phenyl ring of dicarboxylate and two methyl groups of bpz^2–^ linkers, forming short contacts with the coordinated N and O atoms of ligands by its hydrogen (C_R22_···N 3.46–3.58 Å, C_R22_···O 3.61–3.72 Å for **1–3**, Fig. S8†).

Following the increase in R22 loading, the enthalpies of **1** and **2** slowly decrease to *ca.* 30 kJ mol^–1^ and then increase back to *ca.* 32–33 kJ mol^–1^. In contrast, the enthalpy profile of **3** is significantly undulating as indicated by its isotherm shape, which reaches a maximum of 35.5 kJ mol^–1^ at 0.15 g g^–1^ and then decreases to 20.4 kJ mol^–1^ at higher coverage (>0.31 g g^–1^). PXRD patterns of **3** in R22 gas at 1 bar and in air were compared (Fig. S9†), which shows a change in relative peak intensities instead of peak positions. This phenomenon indicates that the coordination framework of **3** keeps unchanged and the undulating adsorption enthalpy profile is caused by rearrangement of the adsorbed adsorbate, being similar with some PCPs.^17^

**Fig. 5:**
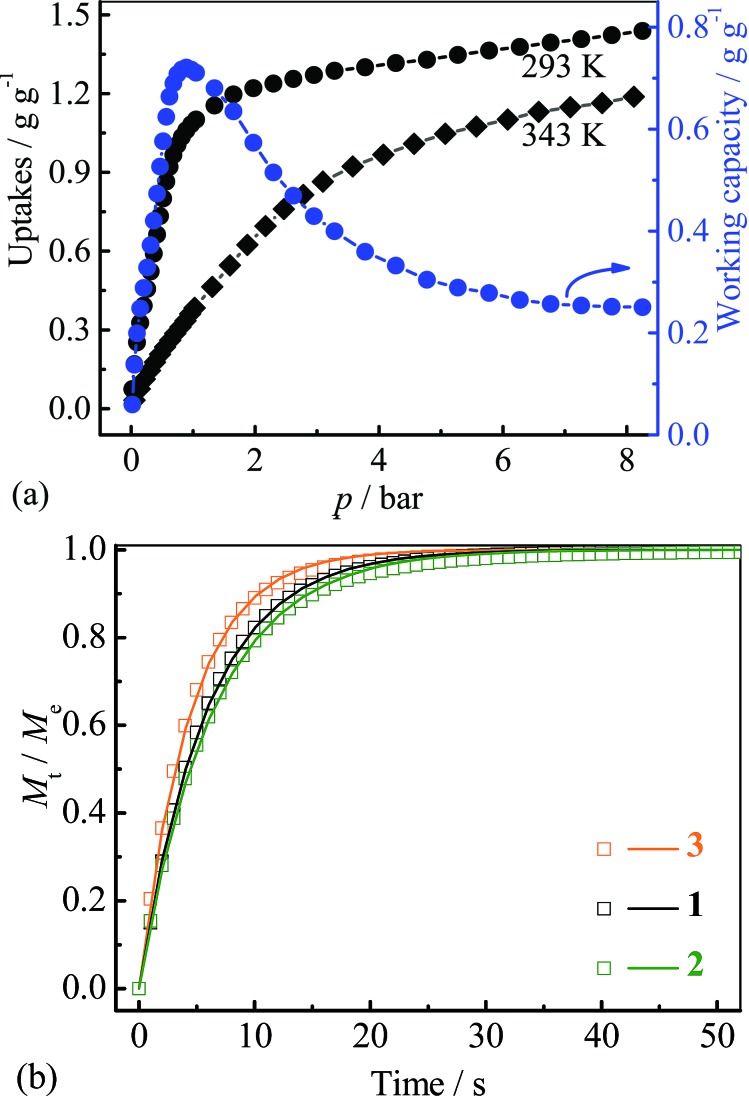
(a) High pressure adsorption isotherms and corresponding uptake difference for **3**, (b) kinetic profiles of R22 adsorption at 313 K for **1–3**.

Since the saturated vapor pressure of R22 at ambient temperatures is sufficiently high (>1 bar), corresponding cold production can be realized at high pressure. To evaluate the performance at such conditions, high-pressure R22 adsorption isotherms for **3** were measured at 293 and 343 K (Fig. 5a). The uptakes at 8 bar are 1.43 g g^–1^ (0.93 g cm^–3^) at 293 K and 1.18 g g^–1^ (0.77 g cm^–3^) at 343 K, respectively. These values are similar to the highest uptakes achieved by some large-surface-area activated carbons (*e.g.* Maxsorb III, surface area: 3140 m^2^ g^–1^, uptakes: 2.10 g g^–1^ or 0.65 g cm^–3^ at 298 K),^46^ and higher than that of MIL-101 (about 1.27 g g^–1^ or 0.79 g cm^–3^ at 298 K) (Table S2†).^32^ Based on the isotherms measured at 293 and 343 K, the highest working capacity was estimated to be 0.72 g g^–1^ or 0.47 g cm^–3^ at 0.9 bar, which is higher than that of Maxsorb III (<0.62 g g^–1^ or 0.19 g cm^–3^) at similar conditions.^46^ The working capacity of **3** gradually decreases to 0.71 g g^–1^ or 0.46 g cm^–3^ at 1 bar, highlighting the good performance at higher working pressures. To ensure the regenerability of **3**, R22 adsorption–desorption cycling measurements were further performed at 293 K, and there was no noticeable loss in adsorption capacity after 8 cycles (Fig. S10†).

Kinetics of R22 adsorption at ambient conditions for **1–3** were analysed (Fig. 5b and S11†). The adsorption–desorption at 313 K can reach equilibrium within 50 seconds, which is significantly faster than that on Maxsorb III (600–1200 s).^50^ The diffusion coefficients were calculated by linear driving force model^51^ to be 5.6, 5.1 and 7.3 × 10^–7^ cm^2^ s^–1^ for **1–3**, respectively (Fig. S11 and 12†, Table 1), being two-order higher than those of Maxsorb III (2.5–5.1 × 10^–9^ cm^2^ s^–1^).^50^ The fast diffusion of R22 in **1–3** should be attributed to the highly uniform pore in the crystalline adsorbents. It can be seen that the larger diffusion coefficients are associated with the compound with larger pore sizes. The high diffusivity of refrigerant in adsorbent is beneficial for rapid adsorption heating and cooling processes, improving the response speed of the system. Interestingly, the pellet form of **1–3** (obtained by simple compressing at about 5 MPa) showed only slightly decreased R22 adsorption kinetics (–6.8%, –4.9% and –2.0%, Fig. S11†) compared with those of the powder form, which is beneficial for practical applications.

## Conclusions

In summary, we demonstrated that highly porous PCPs can show large fluorocarbon adsorption capacity, high diffusivity and good regenerability, which can be candidate adsorbents in heat transformation cycles. Noteworthily, the pore size and/or porosity can impact the sorption performance, demonstrating tunable sorption performance can be obtained by the rational design of PCPs. These results may inspire future design and fabrication of novel adsorbent materials for heat transformation systems.
