# Supplementary material for: Tuning fluorocarbon adsorption in new isoreticular porous coordination frameworks for heat transformation applications
Source: Chem Sci. 2015 Feb 18;6(4):2516–21. doi: 10.1039/c4sc03985h (PMC5647857; doi:10.1039/c4sc03985h)
Supplement: Supplementary file 1 [file SC-006-C4SC03985H-s001.pdf]

Electronic Supplementary Material (ESI) for Chemical Science.
This journal is © The Royal Society of Chemistry 2015

Electronic Supplementary Information

Tuning fluorocarbon adsorption in new isoreticular porous coordination frameworks for heat transformation applications

Rui-Biao Lin, Tai-Yang Li, Hao-Long Zhou, Chun-Ting He, Jie-Peng Zhang* and Xiao-Ming Chen

MOE Key Laboratory of Bioinorganic and Synthetic Chemistry, School of Chemistry and Chemical Engineering, Sun Yat-Sen University, Guangzhou 510275, China.

* E-mail: zhangjp7@mail.sysu.edu.cn

Materials and Methods

Single-Crystal X-ray Diffraction Analyses

Gas Sorption Measurements

Synthesis

GCMC simulations

Fig. S1. Cubelike cavities in **1**–**3**

Fig. S2. PXRD patterns of **1**–**3**

Fig. S3. Thermogravimetry curve of **1·EtOH**, **2·EtOH** and **3·EtOH**

Fig. S4. Pore size distribution for **1**–**3**

Fig. S5. Comparison of R22 adsorption isotherms for **1**–**3**

Fig. S6. Pressure-dependent uptake difference profiles for **1**–**3**

Fig. S7. Adsorption enthalpies of R22 for **1**–**3**

Fig. S8. Preferential R22 location in **1**–**3** obtained from GCMC calculations

Fig. S9. Comparison of the PXRD patterns of **3** in air and R22

Fig. S10. Repeatability of R22 adsorption for **3**

Fig. S11. Exponential fitting of the kinetic profiles of R22 adsorption for **1**–**3**

Fig. S12. Optical images of **1**–**3**

Table S1. Crystallographic parameters of **2** and **3**.

Table S2. Comparison of R22 sorption performance for **1**–**3**

Materials and Methods. The organic ligand H_2bpz is synthesized according to the literature (*J. Chem. Soc.* **1957**, 3997-4003). Other reagents were commercially available and used without further purification. Elemental analyses were performed with an Elementar Vario EL analyzer. Infra-red (IR) spectra were recorded on a Bruker TENSOR 27 FT-IR spectrometer in the 400-4000 cm^{-1} region with KBr pellets. Powder X-ray diffraction (PXRD) patterns were collected on a Bruker D8-Advance diffractometer with Cu Kα radiation and a LynxEye detector. Thermogravimetric analyses (TGA) were performed on a TA Q50 thermogravimetric analyzer with a heating rate of 10 °C/min in nitrogen flow.

Single-Crystal X-ray Diffraction Analyses. The single crystal data of **2·g** were collected at 223 K on a Bruker SMART APEX CCD area-detector diffractometer (Mo Kα). Absorption corrections were applied by using multiscan program *SADABS* [G. M., Sheldrick, *SADABS 2.05*. 2002, University Göttingen: Göttingen, Germany]. The single crystal data of **3·g** were collected at 273 K on a Rigaku XtaLAB P300DS diffractometer (Cu Kα). Absorption corrections were applied by using accompanying program *REQAB* [R. Jacobson, *REQAB*, 1998, Molecular Structure Corporation, The Woodlands, Texas, USA]. The structures were solved with direct methods and refined with a full-matrix least-squares technique with the *SHELXTL* program package [*SHELXTL 6.12*. 2000, Bruker Analytical Instrumentation: Madison, WI]. All non-hydrogen atoms were refined anisotropically except the guest molecules. The organic hydrogen atoms were generated geometrically. As the very disordered solvent molecules could not be modelled, *SQUEEZE/PLATON* procedures were used. Crystal data and details of data collection and refinements are summarized in Table S1. CCDC 1031873−1031874 contains the supplementary crystallographic data for this paper. These data can be obtained free of charge from the Cambridge Crystallographic Data Centre via www.ccdc.cam.ac.uk/data_request/cif.

Gas Sorption Measurements. The sorption isotherms for N_2, R22 were measured with automatic volumetric adsorption apparatuses (BELSORP-HP and Micromertics ASAP 2020M). The as-synthesized sample (ca. 100−200 mg) was placed in the sample holder and dried for 8 h at 393 K to remove the remnant solvent molecules prior to measurements. The temperatures were controlled by a liquid-nitrogen bath (77 K), an ice−water bath (273 K), or a water bath (283, 293, 298, 313 and 343 K).

Due to the limitation of the instrument, the highest measurement temperature and pressure are 343 K and 8.5 bar, respectively.

Synthesis. $[Zn_4O(bpz)_2(bdc)]\cdot$guest (**1·g**, MAF-X10). A mixture of H_2bdc (0.062 g, 0.375 mmol), H_2bpz (0.143 g, 0.75 mmol), $Zn(NO_3)_2\cdot6H_2O$ (0.446 g, 1.5 mmol), N,N-dimethylformamide (DMF, 30 mL), and EtOH (20 mL) was placed in a Teflon-lined stainless steel vessel (100 mL) and heated at 120 ^oC for 72 h, and then it was cooled to room temperature at a rate of 5 $^oC\ h^{-1}$. The resulting colorless block polycrystals of **1·g** were isolated by decanting and treated with ethanol and dried under vacuum (yield 408 mg, 93%). Elemental analysis: Calcd for $C_{28}H_{30}Zn_4N_8O_6$ ($[Zn_4O(bpz)_2(bdc)]\cdot H_2O$): C 40.22, H 3.62, N 13.40. Found: C 40.40, H 3.58, N 13.49%. IR (KBr): 3421m, 2968m, 2923m, 1579vs, 1549vs, 1502m, 1429m, 1381vs, 1134m, 1093w, 1053s, 883w, 812m, 757m, 704w, 553m, 516m cm^{-1}.

$[Zn_4O(bpz)_2(ndc)]\cdot$guest (**2·g**, MAF-X12). This compound was prepared by a similar procedure as **1·g**, except that H_2bdc was replaced by H_2ndc (0.081 g, 0.375 mmol). The resulting colorless block polycrystals of **2·g** were isolated by decanting and treated with ethanol and dried under vacuum (yield 221 mg, 49%). Single crystals for X-ray single-crystal diffraction were prepared by heating a solution of H_2ndc (0.005 g, 0.025 mmol), H_2bpz (0.010 g, 0.05 mmol), and $Zn(NO_3)_2\cdot6H_2O$ (0.030 g, 0.10 mmol) in a mixed solvent of DMF, N-methyl-2-pyrrolidone and iPrOH (v/v/v = 1/1/1, 9 mL) in a 12-mL Teflon-lined stainless steel vessel at 120 ^oC for 72 h, then the mixture was cooled to room temperature at a rate of 5 $^oC\ h^{-1}$ to give colorless block single crystals (18 mg, yield 58%). Elemental analysis: Calcd for $C_{38}H_{56}Zn_4N_8O_{12}$ ($[Zn_4O(bpz)_2(ndc)]\cdot4H_2O\cdot3EtOH$): C 42.32, H 5.23, N 10.39. Found: C 42.35, H 5.01, N 10.16%. IR (KBr): 3403m, 2966m, 2923m, 1566vs, 1508s, 1422vs, 1366vs, 1263m, 1210w, 1135m, 1052vs, 980w, 869w, 794m, 705w, 666w, 562m, 518m, 465w cm^{-1}.

$[Zn_4O(bpz)_2(bpdc)]\cdot$guest (**3·g**, MAF-X13). A solution of H_2bpdc (0.006 g, 0.025 mmol), H_2bpz (0.010 g, 0.05 mmol), and $Zn(NO_3)_2\cdot6H_2O$ (0.030 g, 0.10 mmol) in a mixed solvent of DMF, N,N-diethylformamide and iPrOH (v/v/v = 4/1/3, 8 mL) in a 12-mL Teflon-lined stainless steel vessel at 120 ^oC for 72 h, and then the mixture was cooled to room temperature at a rate of 5 $^oC\ h^{-1}$ to give colorless flake single crystals (23 mg, yield 64%). Elemental analysis: Calcd for $C_{35}H_{40.2}Zn_4N_8O_{8.1}$

($[Zn_4O(bpz)_2(bpdc)]\cdot 2.6H_2O\cdot 0.5EtOH$): C 43.60, H 4.20, N 11.62. Found: C 43.56, H 4.05, N 11.49%. IR (KBr): 3292m, 2957m, 2916m, 1608vs, 1548m, 1497m, 1424m, 1336vs, 1173w, 1131m, 1052s, 836m, 772s, 703w, 673w, 546m, 523m, 460 w cm^{-1}.

GCMC simulations. All the GCMC simulations in the MS modeling 5.0 package [*Accelrys*, Materials Studio Getting Started, release 5.0, Accelrys Software, Inc., San Diego, CA, 2009]. The framework and the individual R22 molecules were considered to be rigid. Partial charges for atoms of **1**–**3** were derived from QEq method and QEq_neutral1.0 parameter. The simulations were carried out at 298 K, adopting the Fixed Loading task, Metropolis method in Sorption module and the universal forcefield (UFF). The partial charges on the atoms of R22 molecules were C 0.171e, H 0.063e, Cl −0.046e and F −0.094e (where $e = 1.6022\times10^{-19}$ C is the elementary charge), respectively. The interaction energy between R22 and framework were computed through the Coulomb and Lennard-Jones 6-12 (LJ) potentials. The cutoff radius was chosen as 18.5 Å for the LJ potential and the long-range electrostatic interactions were handled using the Ewald & Group summation method. The loading steps and the equilibration steps were 1×10^6, the production steps were 5×10^6.

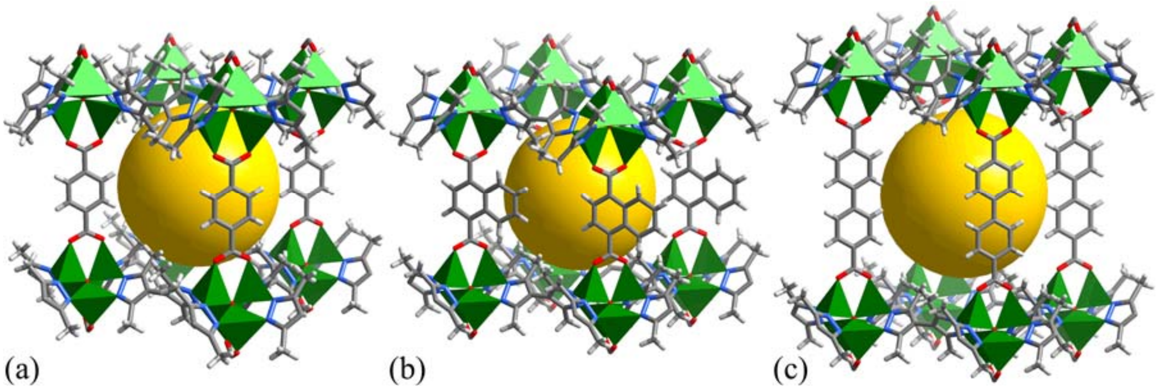

Figure S1. Cubelike cavities in **1** (a), **2** (b) and **3** (c).

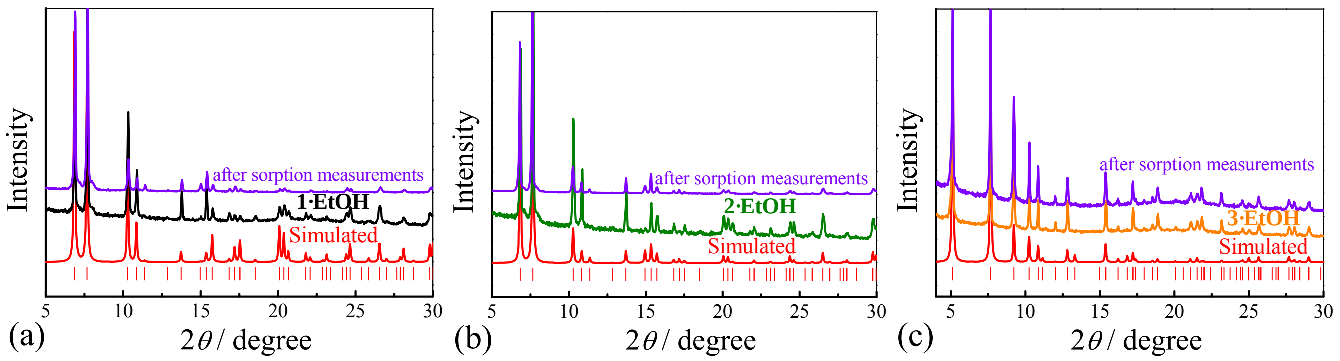

Figure S2. PXRD patterns of **1**, **2** and **3**.

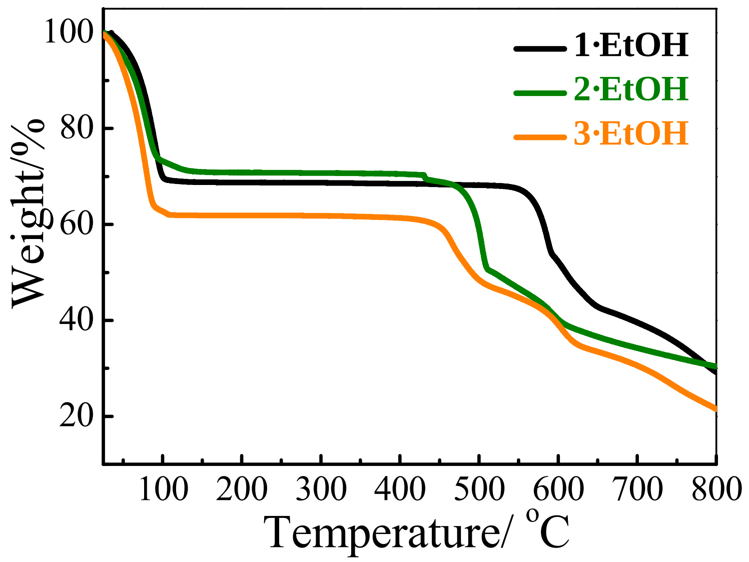

Figure S3. Thermogravimetry curve of **1·EtOH**, **2·EtOH** and **3·EtOH**.

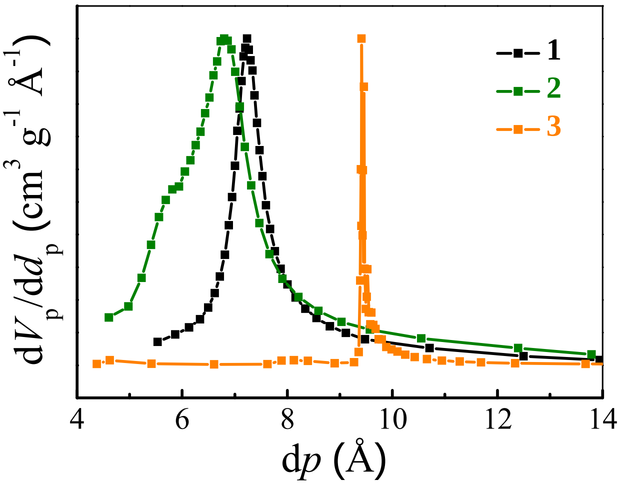

Figure S4. Pore size distribution (Horvath–Kawazoe model) of **1**–**3** calculated from the N_2 adsorption isotherm.

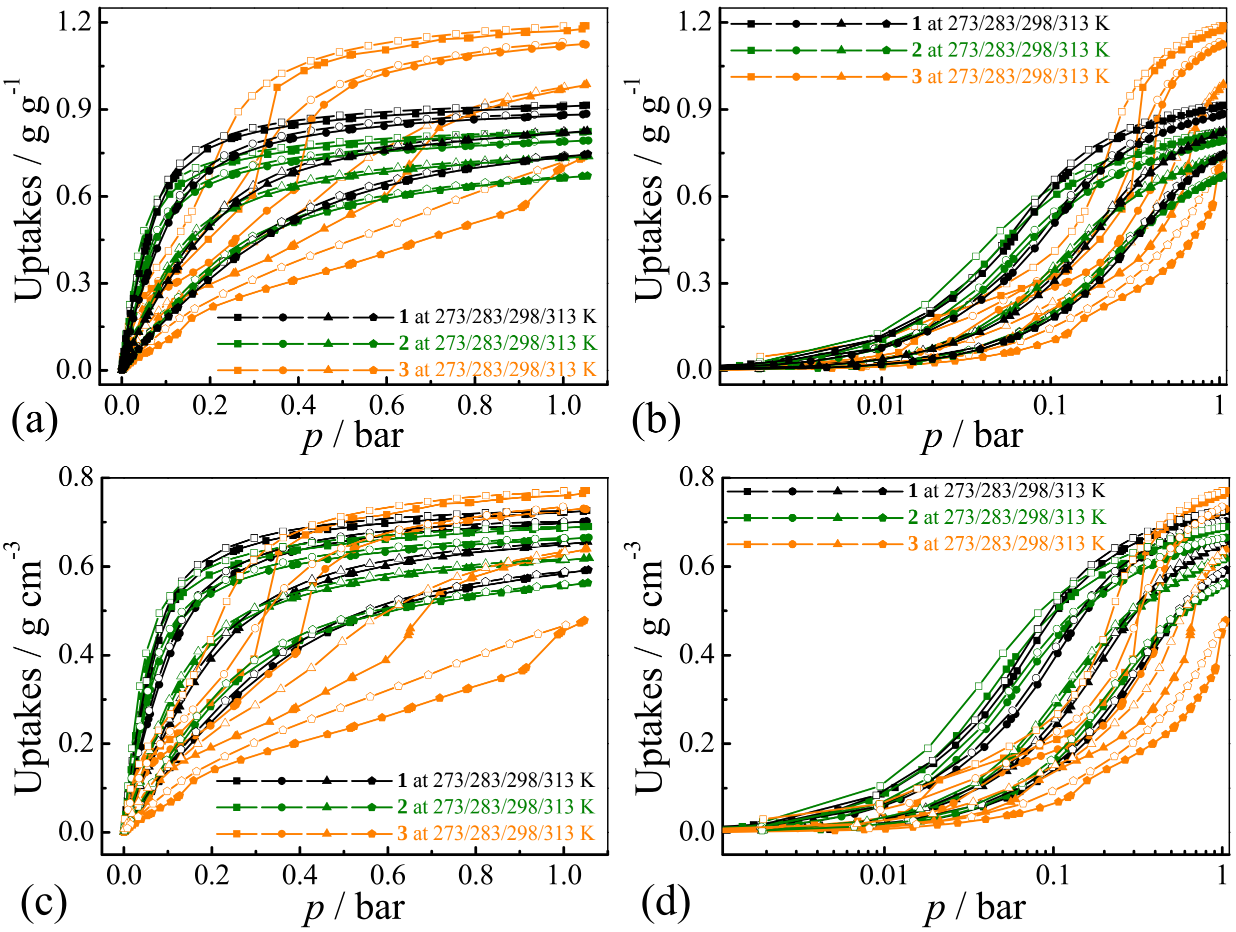

Figure S5. Comparison of R22 adsorption (solid) and desorption (open) isotherms for **1**–**3**. (a) Gravimetric uptakes with linear horizontal axis, (b) gravimetric uptakes with logarithmic horizontal axis, (c) volumetric uptakes with linear horizontal axis and (d) volumetric uptakes with logarithmic horizontal axis.

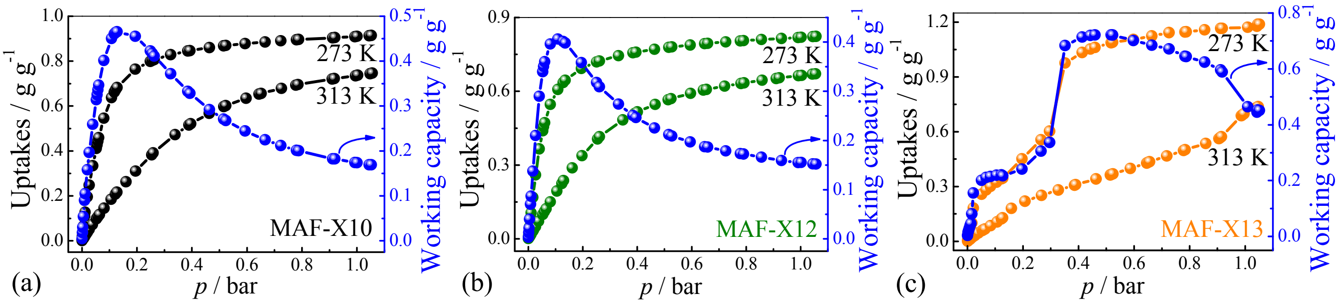

Figure S6. Pressure-dependent uptake difference profiles for **1**–**3** between adsorption isotherms measured at 273 and 313 K.

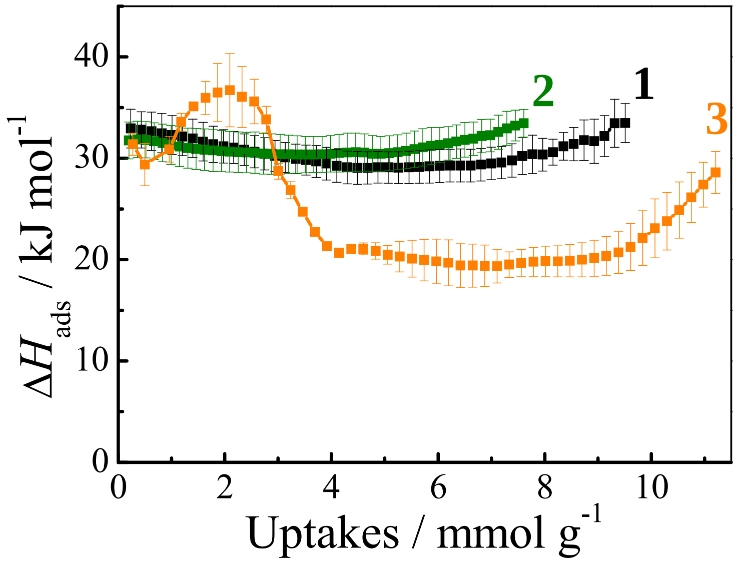

Figure S7. Adsorption enthalpies of R22 for **1**, **2** and **3** calculated by Clausius−Claperyron equation.

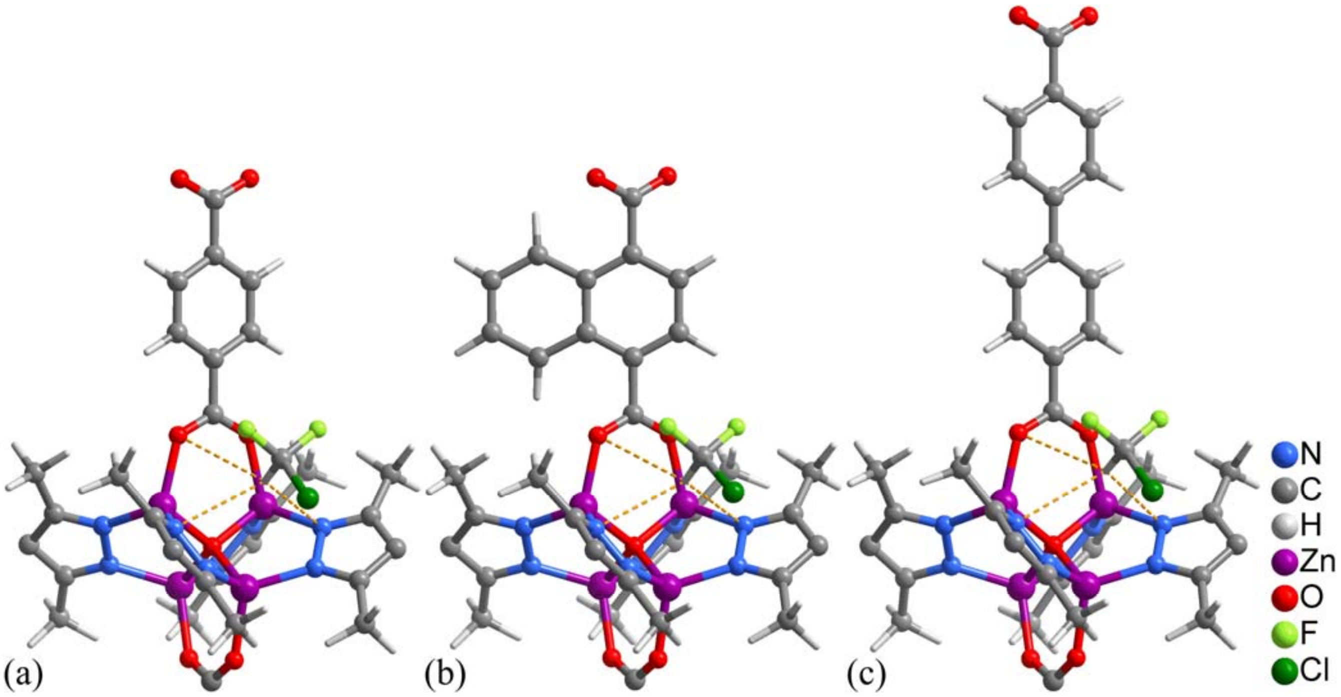

Figure S8. Preferential R22 location in (a) **1**, (b) **2**, and (c) **3** obtained from GCMC calculations. For **1**, $C_{R22}\cdots N$ 3.46 and 3.52 Å, $C_{R22}\cdots O$ 3.65 Å; For **2**, $C_{R22}\cdots N$ 3.48 and 3.47 Å, $C_{R22}\cdots O$ 3.61 Å; For **3**, $C_{R22}\cdots N$ 3.55 and 3.58 Å, $C_{R22}\cdots O$ 3.72 Å

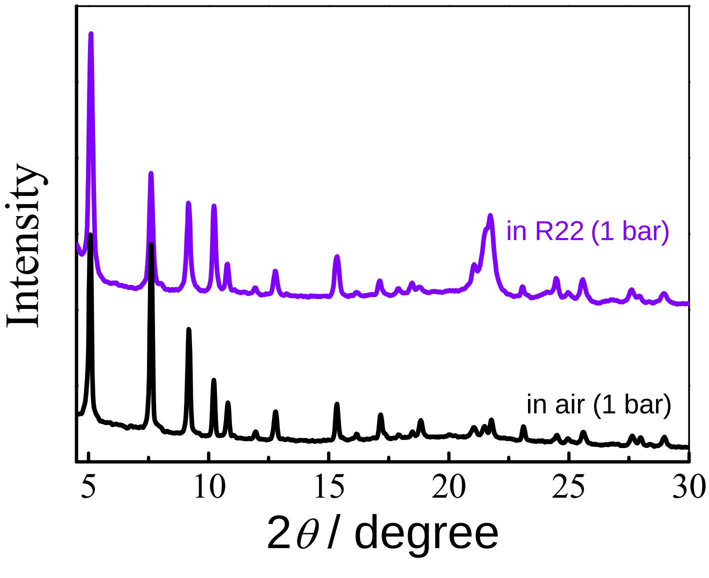

Figure S9. Comparison of the PXRD patterns of **3** in air and R22 at 273 K.

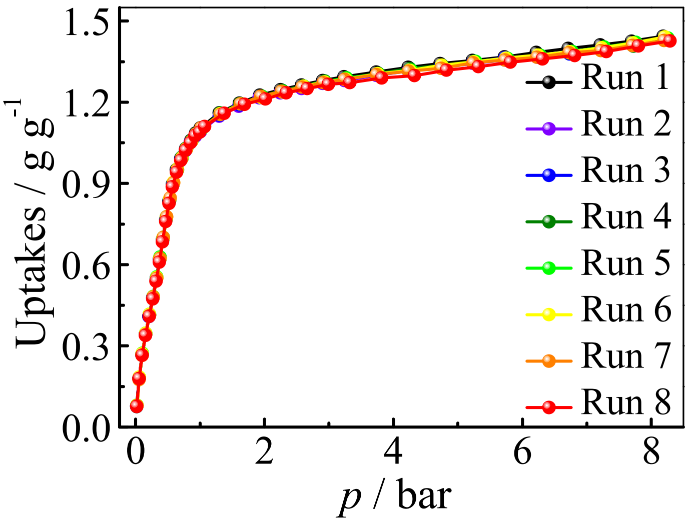

Figure S10. Repeatability of R22 adsorption at 293 K for **3**.

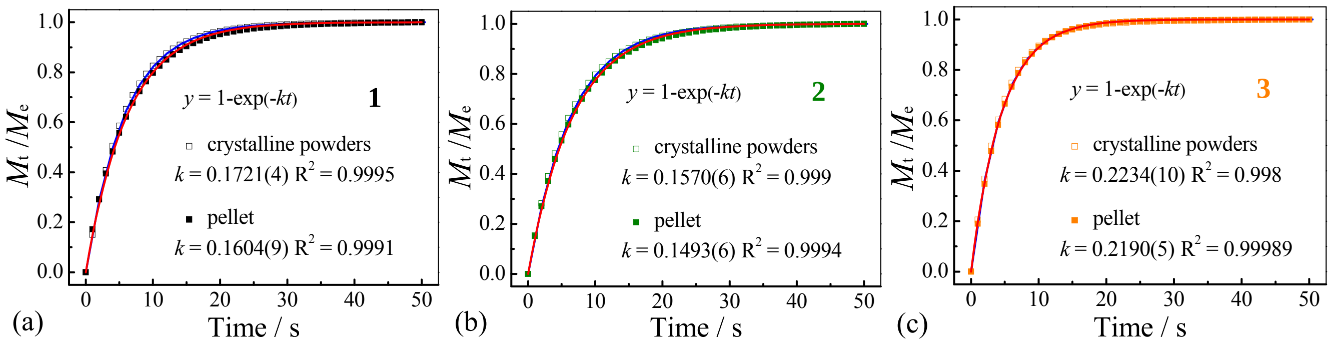

Figure S11. Exponential fitting of the kinetic profiles of R22 adsorption at 313 K for (a) **1**, (b) **2**, and (c) **3** in crystalline powders (open) and pellets (solid) form, respectively (lines represent the exponential fitting).

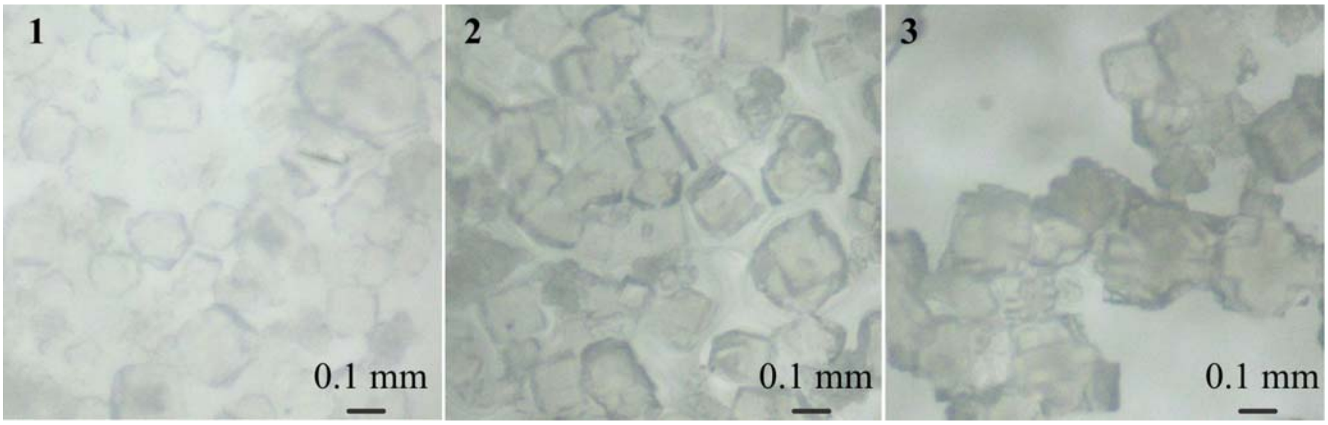

Figure S12. Optical images of (a) **1**, (b) **2**, and (c) **3** (the average particles radii are estimated to be ca. 0.007 cm for the three compounds). The diffusion coefficient $D_{R22} = k{*}r_c^2/15$=0.1721 s^{-1} × (0.007 cm)^2 ÷ 15= 5.6 × 10^{-7} cm^2/s for **1**, while 5.1 × 10^{-7} and 7.3 × 10^{-7} cm^2/s for **2** and **3**, respectively.

Table S1. Crystallographic parameters of **2** and **3**.

Complex	**2**	**3**
Formula	$C_{32}H_{30}N_8O_5Zn_4$	$C_{34}H_{32}N_8O_5Zn_4$
Formula weight	868.12	894.16
Temperature (K)	223(2)	273(2)
Crystal system	Tetragonal	Tetragonal
Space group	$P4_2/mcm$	$P4_2/mcm$
a/Å	11.5382(14)	11.517(3)
c/Å	25.813(3)	34.474(11)
V/Å^3	3436.4(7)	4573(2)
Z	2	2
D_c /g cm^{-3}	0.839	0.649
reflns coll.	12520	13199
unique reflns	1858	1819
R_{int}	0.0554	0.0780
R_1 $[I > 2\sigma(I)]$[a]	0.0524	0.0748
wR_2 (all data)[b]	0.1609	0.1958
GOF	1.025	1.095

[a] $R_1=\Sigma||F_o| - |F_c||/\Sigma|F_o|$. [b] $wR_2 =[\Sigma w(F_o^2 - F_c^2)^2/\Sigma w(F_o^2)^2]^{1/2}$.

Table S2. Comparison of R22 sorption performance for **1**, **2** and **3**.

Species	Density (g/cm^3)	Ambient-pressure				High-pressure	
		273 K		298 K		Room temperature (5 bar)	
		g/g	g/cm^3	g/g	g/cm^3	g/g	g/cm^3
**1**	0.794	0.91	0.72	0.82	0.65	/	/
**2**	0.839	0.82	0.69	0.73	0.62	/	/
**3**	0.649	1.17	0.76	0.97	0.63	1.34	0.87
MIL-101[a]	0.620	/	/	0.85	0.53	1.27	0.79
Maxsorb III[b]	0.310	/	/	/	/	1.90	0.59

[a]: data from [P. K. Thallapally et al., *Nat. Commun.* **2014**, *5*, 4368]; [b]: data from [B. B. Saha et al, *Int. J. Refrig.* **2009**, *32*, 1563-1569].
